# Intertidal and nearshore Nereididae (Annelida) of the Falkland Islands, southwestern Atlantic, including a new species of *Gymnonereis*

**DOI:** 10.3897/zookeys.427.7296

**Published:** 2014-07-22

**Authors:** Teresa Darbyshire

**Affiliations:** 1 Amgueddfa Cymru–National Museum Wales, Department of Natural Sciences, Cathays Park, Cardiff CF10 3NP, Wales, U.K.

**Keywords:** Taxonomy, paragnaths, Polychaeta, *Eunereis*, *Neanthes*, *Perinereis*, *Platynereis*

## Abstract

The intertidal and nearshore Nereididae of the Falkland Islands are detailed and a new species of *Gymnonereis* described. The new species, *Gymnonereis tenera* **sp. n.**, is the first record of the genus for the Falkland Islands. It is, so far, only known from a few intertidal locations in fine and muddy sands. Main distinguishing characters are: jaw teeth absent (in adults), 3 papillae in Area V–VI, falcigers absent, second ventral cirrus present throughout. *Nereis atlantica* McIntosh, 1885, known only from the description of a single specimen and one doubtful record from the Falkland Islands, is reviewed and transferred to *Perinereis* on the basis of the presence of shield-shaped bars in Area VI of the proboscis and the absence of notopodial falcigers. A key to all seven species discussed is provided.

## Introduction

The Nereididae is one of the largest polychaete families (Hutchings et al. 2000) and, intertidally, one of the most widespread and frequently encountered (Wilson 2000). Although not much literature exists on the intertidal polychaetes of the Falkland Islands, at least one species of Nereididae is recorded in each of the three papers (Pratt 1898, 1901; Fauvel 1916) published. Other records of Falkland Islands Nereididae have been from Antarctic/Southern Ocean research cruises that have also taken samples among and around the islands (Ramsay 1914; Monro 1930, 1936; Hartman 1953, 1967).

In all, eight species of nereidid, in six genera, have previously been recorded from stations listed as being within the Falkland Islands region. However, two of these species, *Platynereis australis* (Schmarda, 1861) and *Platynereis magalhaensis* Kinberg, 1865 have been controversially synonymized (e.g. Benham 1909, 1921; Hutchings and Reid 1990) or kept separate (e.g. Fauvel 1916; Augener 1932; Hartman 1953, 1964) many times historically. Most recently, Read (2007) maintained both names pending investigation of the epitokous stage of *Platynereis magalhaensis* to help resolve the issue. All but one record (Ramsay 1914) of *Platynereis* for the islands are as *Platynereis magalhaensis* and this name is therefore retained in this paper with a discussion of the current situation. *Platynereis australis* is considered unlikely to occur around the islands, hence a description is not included.

Only species that have previously been recorded from Falkland Islands samples taken in less than 30 m (where diving and shallow survey work are most likely to take place) are considered in this paper. For this reason, *Nicon maculata* Kinberg, 1865 is also excluded as it has not been recorded from less than 129 m in the area (Monro 1936; Hartman 1953, 1967). *Eunereis patagonica* (McIntosh, 1885), *Nereis atlantica* McIntosh, 1885 and *Nereis eugeniae* (Kinberg, 1865) were not collected by the survey but are included because they have previously been recorded from shallow depths and could feasibly be recorded from shallow samples taken in the area. The descriptions and reports of each species are considered and details specific to the Falkland Islands reported. *Nereis atlantica* McIntosh, 1885 is reviewed and newly transferred to *Perinereis* Kinberg, 1865.

Most of the nereidids collected were found in mainly coarse or hard habitats, however, a new species of *Gymnonereis* Horst, 1919,a genus not previously recorded from Falkland Island waters, was identified from a small number of localities where it was almost entirely confined to intertidal, fine and muddy sands. *Gymnonereis* is a small genus of only six recognized species: *Gymnonereis sibogae* Horst, 1918 (type locality: Strait of Makassar, Indonesia), *Gymnonereis crosslandi* Monro, 1933 (type locality: Gorgona Island, Colombian Pacific), *Gymnonereis fauveli* Hartmann-Schröder, 1962 (type locality: San Julián, Argentina), *Gymnonereis phuketensis* Hylleberg & Nateewathana, 1988 (type locality: Andaman Sea, Thailand), *Gymnonereis minyami* Hutchings & Reid, 1990 (type locality: Victoria, South Australia) and *Gymnonereis yurieli* Hutchings & Reid, 1990 (type locality: Queensland, Australia). All members of the genus lack paragnaths, having only soft papillae on the oral ring and all, except *Gymnonereis crosslandi*, exhibit highly vascularized dorsal cirrophores on median chaetigers. The new species is distinguishable from the other members of the genus using combinations of characters detailing the presence or absence of jaw teeth, falcigers and enlarged dorsal cirrophores, the number and distribution of the oral ring papillae, the occurrence of accessory dorsal cirri and the relative lengths of the neuropodial lobes.

A key to the seven species of Nereididae recognized from the near shore (< 30 m) waters of the Falkland Islands is provided.

## Terminology

**Figure 1.**
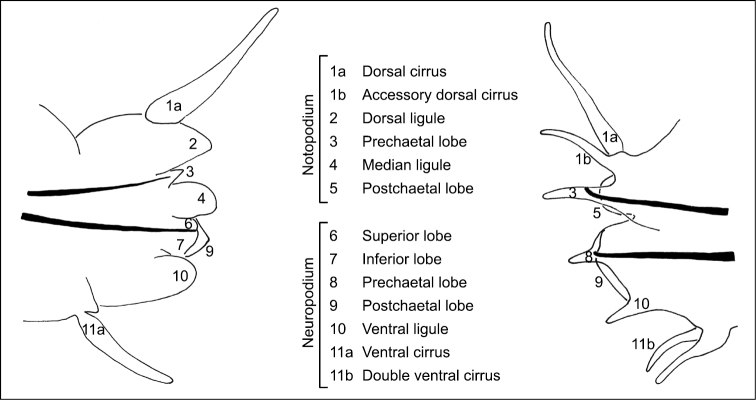
Terminology and diagrammatic representation of **A** a Nereidinae parapodium (modified from Hutchings and Reid 1990) and **B** a Gymnonereidinae parapodium (modified from Hylleberg and Nateewathana 1988), anterior view.

The parapodia of the Gymnonereidinae are more complex than those of the Nereidinae and a diagram is provided in Figure 1 to compare and standardize the terminology used in this paper when describing the different species. In reference to *Gymnonereis*, the terminology used by Hutchings and Reid (1990) has been mostly adopted, with some modification according to Santos et al. (2005), and is detailed further in the Remarks for that section.

The terminology used to describe paragnath forms was reviewed by Bakken et al. in 2009 and has been applied here also. Where necessary, generic diagnoses have been emended to reflect this, with changes highlighted in italics.

## Methods

In 2011 and 2013, intertidal and diving survey work was undertaken around the Falkland Islands, covering the two main islands, East and West Falkland, as well as some of the smaller outer islands. Specimens were collected by a variety of methods on the shore and by SCUBA diving. Intertidal habitats were investigated by digging and collecting specimens by hand, by sieving sediment through a 0.5 mm sieve, turning over rocks and removing attached tubes, splitting open rock crevices and by inspecting algal holdfasts. Sampling while diving involved scraping epifaunal turf off rocks, turning over rocks and removing attached tubes, and taking sediment samples that would later be sieved as above.

Specimens were relaxed with a 7% magnesium chloride solution where possible and then fixed with 4% formaldehyde in seawater. After a period of at least 2 days, animals were rinsed with freshwater and preserved in 80% industrial methylated spirits with 2% propylene glycol added.

Morphological examinations and measurements were made using a Nikon Eclipse E400 binocular microscope and a Nikon Labophot-2 compound microscope. Microscope photographs were taken using AutoMontage™ software.

The holotype and most paratypes of *Gymnonereis tenera* sp. n. are accessioned in the zoological collections of National Museum Wales (NMW.Z). Paratypes are also deposited in the Australian Museum (AM), Natural History Museum, London (NHMUK), National Museum of Natural History, Smithsonian Institution, Washington D.C. (USNM) and the Zoological Museum, Hamburg (ZMH). All other specimens are accessioned in the National Museum Wales collections.

## Taxonomy

### Family Nereididae Blainville, 1818

#### Subfamily Gymnonereidinae Banse, 1977

##### Genus Gymnonereis

Taxon classificationAnimaliaPhyllodocidaNereididae

Horst, 1919

Gymnorhynchus Horst, 1918: 247. — Pre-occupied by *Gymnorhynchus* Rudolphi, 1819; Cestoda (paper cited from Pettibone 1970).
Gymnonereis Horst, 1919: 64. — Pettibone 1970: 234. — Banse 1977: 621–622 (in part).

###### Type species.

*Gymnonereis sibogae* (Horst, 1918) by monotypy

###### Diagnosis

(after Hutchings and Reid 1990). Body elongate, depressed, attenuated posteriorly. Prostomium with frontal margin deeply incised between bases of frontal antennae.

Eversible pharynx with jaws having cutting edge smooth or serrated, with papillae on the oral ring. Notopodia with accessory dorsal cirri attached to dorsal cirrophores in anterior region only, with prechaetal lobes and short, rounded postchaetal lobes. Median segments with dorsal cirrophores greatly elongated and highly vascularized (except in *Gymnonereis crosslandi*) and lacking accessory cirri. Dorsal transverse ridges present or absent. Chaetae homogomph or sesquigomph spinigers and homogomph or sesquigomph falcigers may be present. Chaetae very numerous in anterior chaetigers.

###### Remarks.

Hutchings and Reid (1990) used the term ‘sesquigomph', in a review of Australian Gymnonereidinae, to describe those chaetae that have a 3:2 ratio between the boss and opposing prong of the shaft. Such chaetae were referred to as 'slightly hemigomph' by Fauchald (1977) or 'slightly heterogomph' by Hylleberg and Nateewathana (1988) in their descriptions of *Gymnonereis*. Terminology referring to the additional dorsal (=accessory dorsal) cirrus and ventral (=double ventral) cirri follow that of both Hutchings and Reid (1990) and Santos et al. (2005) in the first instance but only Santos et al. (2005) in the second. Finally, the parapodial projections referred to as ‘prechaetal ligules’ in both Hylleberg and Nateewathana (1988) and Hutchings and Reid (1990) are here termed prechaetal lobes, after Santos et al. (2005), who defined notopodial projections supported by aciculae as lobes and those without aciculae as ligules and found notopodial ligulae to be absent in *Gymnonereis*. This definition has also been applied here to the previously-termed ‘neuropodial prechaetal ligules’, referred to here as neuropodial prechaetal lobes (Fig. 1).

##### Gymnonereis tenera sp. n.

Taxon classificationAnimaliaPhyllodocidaNereididae

http://zoobank.org/66F36C23-ECF2-4F01-A2CF-BB12F84D1894

Figures 2A–I , 9A–B

###### Material examined.

East Falkland: Teal Creek, Stn 35d (51°49.248'S, 058°55.561'W), muddy sand, midshore, holotype (NMW.Z.2011.039.0102), 09.12.2011; Sand Bay, Port Harriet, Stn 34d (51°44.231'S, 058°00.585'W), fine sand, mid–low shore, 11 paratypes (9–NMW.Z.2011.039.0093–0095; 1–USNM 1231433; 1–ZMH p-27694), 08.12.2011; Teal Creek, Stn 35b (51°49.231'S, 058°55.573'W), sandy mud, midshore, 18 paratypes (NMW.Z.2011.039.0096), 09.12.2011; Teal Creek, Stn 35c (51°49.236'S, 058°55.563'W), mud, low shore, 22 paratypes (NMW.Z.2011.039.0097–0101), 09.12.2011; West Falkland: Crooked Inlet, Roy Cove, Stn 55b (51°32.546'S, 060°20.562'W), fine sand, high-midshore, 4 paratypes (1–AM W.46477; 1–NHMUK ANEA2014.31; 2– NMW.Z.2012.082.0001), 30.01.2013; Crooked Inlet, Roy Cove, Stn 55c (51°32.595'S, 060°20.367'W), fine sand, midshore, 2 paratypes (NMW.Z.2012.082.0002), 30.01.2013; Crooked Inlet, Roy Cove, Stn 55d (51°32.664'S, 060°20.255'W), fine sand, low shore, 3 paratypes (NMW.Z.2012.082.0003–0004), 30.01.2013; Crooked Inlet, Roy Cove, Stn 55e (51°32.688'S, 060°20.244'W), fine sand, low shore, 2 paratypes (NMW.Z.2012.082.0005), 30.01.2013.

###### Description.

Holotype complete, 98 mm long, 1.5 mm wide (excluding parapodia; measured at widest part of anterior – approximately chaetiger 8), for 160 chaetigers. Complete paratypes 3–143 mm long, 0.15–2.53 mm wide (excluding parapodia) for 28–166 chaetigers. Description based on observations of the holotype and a dissected paratype (NMW.Z.2011.039.0098) used for illustrations. Variation shown by other paratypes described in later section.

Body depressed dorso-ventrally, widest anteriorly on chaetigers 8–10 (more pronounced in smaller specimens), then mostly uniform in width before tapering posteriorly. Colour pink/orange or grey/white in alcohol with black aciculae. Neurochaetae and subacicular notochaetae dark golden in anterior chaetigers, supracicular chaetae pale amber; all chaetae pale amber from chaetiger 14. Live animals bright red on each side of body, including the parapodia, in region of vascularized, enlarged cirrophores; rest of body often with bright white dorsal bands centrally either side of central blood vessel from end of vascularized cirrophore region, fading in posterior. Where white colouration absent, body transparent, coloured only by visible gut and blood vessels. Methyl green staining of preserved animals shows glandular areas on tips of cirri and parapodial lobes but not on cirrophores or main body. Cuticle very soft when animals alive as well as post-fixation, body breaks easily when handled.

**Figure 2.**
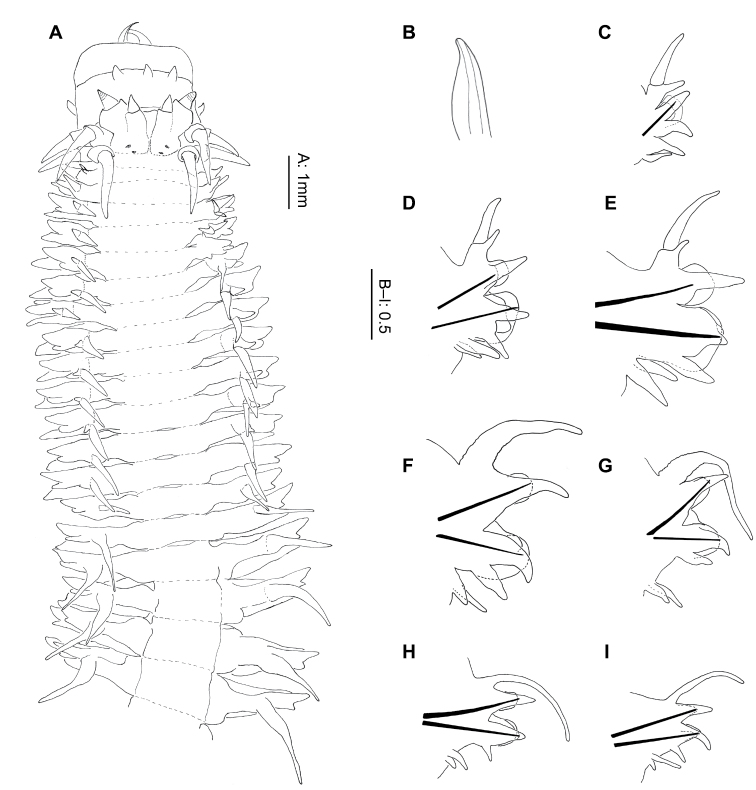
*Gymnonereis tenera* sp. n. (**A** NMW.Z.2011.039.0102 **B–I** NMW.Z.2011.039.0098): **A** holotype, anterior end, dorsal view (right chaetiger 4 aberrant) **B** jaw; C: left parapodium, chaetiger 1, anterior view **D** left parapodium, chaetiger 3, anterior view **E** left parapodium, chaetiger 9, anterior view **F** left parapodium, chaetiger 20, anterior view **G** left parapodium, chaetiger 30, anterior view **H** left parapodium, chaetiger 50, anterior view **I** left parapodium, chaetiger 100, anterior view.

Prostomium with 2 pairs small, black (dark red when alive) eyes, often difficult to discern when preserved (Figs 2A, 9A). Anterior pair smaller, more laterally placed than posterior pair; crescent-shaped with additional small spot in far corners. Posterior pair darker, rounded. Prostomium subrectangular with deep cleft between antennae (Fig. 2A). Palps with large squat palpophores and short triangular palpostyles (0.4 mm long, 0.27 mm wide). Antennae equal length to or slightly longer than palps, more slender in form. Four pairs tentacular cirri, ventral pairs of equal length, 2/3 to 1/2 length of dorsal pairs; 2^nd^ pair dorsal tentacular cirri marginally longer than 1^st^ pair, reaching to chaetiger 4.

Peristomium dorsally more narrow than following segments. Jaws with smooth edges, teeth absent (Fig. 2A–B). Oral ring with triangular papillae arranged as follows (Figs 2A; 9A, B): Area V–VI = 3, VII–VIII = 7; maxillary ring bare.

Chaetigers 1–2 uniramous (Fig. 2C), single black acicula, tip curved, just emergent. Subsequent chaetigers all biramous (Fig. 2D–I), notoacicula not emergent, neuroacicula thicker, emergent anteriorly only up to around chaetiger 50. Dorsal cirri of chaetigers 1–12 with accessory dorsal cirrus (Fig. 2A, C–E), up to 1/3 length of main cirrus, appearing as extension to cirrophore rather than dorsal cirrus. From chaetigers 16–52 (Fig. 2F–G), dorsal cirrophores expanded and vascularized, although start and end of region difficult to define. Remaining chaetigers with dorsal cirrus long, narrow, tapering (Fig. 2H–I).

Double ventral cirri present throughout (Fig. 2C–I), branches unequal, ventral branch reducing in size posteriorly. Dorsal branch 1.5 times as long as ventral branch in anterior region, 4–5 times as long posteriorly.

Chaetiger 1 (Fig. 2C), neuroacicular papilla small, rounded, posterior and slightly dorsal to digitiform prechaetal lobe. Postchaetal lobe broad, rounded, approximately 2/3 length of prechaetal lobe. Acicular lobe similar shape to postchaetal lobe, approximately 1/2 length. Ventral neuropodial ligule of same size and shape as prechaetal lobe.

Chaetiger 3 (Fig. 2D) with basally swollen, digitiform notopodial prechaetal lobe twice as long as broadly rounded notopodial postchaetal lobe; acicular lobe 1/4 length of latter. Notochaetae in 2 unequal bundles, arranged as a smaller semicircle above and larger semicircle below the notopodial prechaetal lobe. Neuropodium as for chaetiger 1, ventral ligule of same size and shape as neuropodial prechaetal lobe. Neurochaetae in 2 semicircular fascicles of greater density than notochaetae. Superior fascicle arranged around neuroacicular papilla with larger, inferior bundle ventral and posterior to neuropodial prechaetal lobe. Arrangement continues to start of vascularized cirrophores then number of chaetae reduces posteriorly, becoming bundles rather than semicircles. Greatest density of chaetae occurs in chaetigers 6–8.

Posteriorly, neuropodial prechaetal lobe reducing in size, ventral ligule even more so. Neuropodial postchaetal lobes also decrease in size proportionately, becoming more conical.

Noto- and neurochaetae consist of both homogomph and sesquigomph spinigers throughout, no falcigers observed. Accurate numbers of chaetae and distribution of homogomph versus sesquigomph chaetae on anterior chaetigers difficult to identify due to density.

**Table 1.** Approximate chaetal counts of *Gymnonereis tenera* sp. n. (paratype, NMW.Z.2011.039.0098).

Chaetiger	Notochaetae	Neurochaetae
1	0	30
3	20	90
9	39	108
20	19	40
30	20	40
50	10	12
100	8	19

No dorsal flaps connecting chaetigers. Transverse, faintly defined ridges present from chaetiger 11–16.

Pygidium with anus terminal; 2 long cirri ventral to anus. Anal cirri of similar shape to dorsal cirri on body, 1.2 mm long.

Eggs found in 2 specimens, spherical, 120–130 µm diameter.

###### Variation.

Most characters varied with number of chaetigers and continued to change as the number increased. Accessory dorsal cirri were not observed on animals with less than 95 chaetigers (unless regenerating) although they were absent in one specimen of 103 chaetigers (62 complete specimens examined; 27 with less than 95 chaetigers, 35 with 95 or more chaetigers). As chaetiger number increases, additional anterior dorsal cirri have accessory cirri, with animals of more than 160 chaetigers with accessory dorsal cirri as far as chaetigers 10–14. The variation in this character means that it should not be used as diagnostic for the species on its own but only in conjunction with other characters.

The faint transverse ridges connecting parapodia were mostly visible from chaetiger 11 to 15 or 16 but were occasionally observed as far back as chaetiger 20 on the largest specimens.

Determination of the start and end of the expanded cirrophores was difficult, particularly the former, as the transition was not as abrupt as described for some species. The region generally occurred from around chaetigers 11–18 and continued to chaetigers 22–51 over the range of body sizes observed.

Presence and number of the oral papillae did not vary with size although papillae were occasionally lost and a single specimen was identified with 4 papillae in Area V–VI. Relative length of tentacular cirri was also stable with the longest cirri always reaching to chaetiger 4 in all body sizes.

Although jaw teeth were absent in the majority of specimens, juveniles of less than 80 chaetigers (jaws of 26 specimens were examined including 12 juveniles of 33–80 chaetigers in size) were found to have 4–5 small teeth on each jaw with jaws in larger animals becoming more roughly crenated until the largest jaws appeared almost completely smooth.

###### Etymology.

The specific name *tenera* is derived from the latin adjective *tener* meaning ‘soft, delicate’, referring to the very soft nature of the worm when alive and its fragility when handled.

###### Habitat.

Found intertidally from mid to low shore in soft, fine, sand or mud sediments.

###### Remarks.

With 3 papillae in Area V–VI of the oral ring and the absence of jaw teeth, *Gymnonereis tenera* sp. n. can be distinguished from all other *Gymnonereis* species except for *Gymnonereis sibogae* and *Gymnonereis phuketensis*. *Gymnonereis minyami* and *Gymnonereis yurieli* both have jaw teeth and only 1 papilla in each of Areas V and VI. *Gymnonereis crosslandi* and *Gymnonereis fauveli* both lack jaw teeth but *Gymnonereis crosslandi* has only 1 papilla in each of Areas V and VI, accessory dorsal cirri in only chaetigers 1 and 2 (chaetiger 1 to 12 or further in *Gymnonereis tenera* sp. n.) and no enlarged dorsal cirrophores, whilst *Gymnonereis fauveli* has 5 papillae in Area V–VI and accessory dorsal cirri from chaetiger 3 (as opposed to chaetiger 1 in the new species).

*Gymnonereis tenera* sp. n. is most similar to both *Gymnonereis sibogae* and *Gymnonereis phuketensis* and can only be distinguished from each of these through combinations of characters. Although Hutchings and Reid (1990) listed *Gymnonereis sibogae* as having sesquigomph falcigers, Horst (1918), in his original description, actually stated that “the neuropodial fascicle does not contain true setae falcigerae, but instead of these some faintly heterogomph setigerous bristles, with a short, lanceolate terminal piece”, although his figures of the species (Horst 1924) did not illustrate this. Pettibone (1970) re-investigated and drew all of Horst’s specimens and in her detailed description of the first two chaetigerous segments stated that “a few lower neurosetae of some anterior setigers may have blades which end bluntly” and this was figured accordingly (Pettibone 1970, fig. 30c–e). No such short, blunt chaetae were observed on any specimens of *Gymnonereis tenera* sp. n. A more consistent character is that of the length of the anterior, neuropodial prechaetal lobe. In *Gymnonereis tenera* sp. n., this lobe is consistently longer than both the neuropodial acicular and postchaetal lobes and of a similar length to the ventral ligule. In *Gymnonereis sibogae*, the neuropodial prechaetal lobe (termed the prechaetal ligule by Pettibone 1970) is as long as or shorter than the postchaetal lobe and shorter than the ventral ligule for at least the first nine chaetigers (Horst 1924, pl. XXX, fig. 1; Pettibone 1970, fig. 30c–d, fig. 31a,d,e,f, fig. 33b), thereafter becoming only slightly longer. Unfortunately, all of Horst’s specimens were incomplete with only 36–56 segments and the species does not appear to have been reported since, making further determination of differences between the two species difficult.

Apart from the character of presence or absence of jaw teeth, the new species is also very similar to *Gymnonereis phuketensis*, although juveniles of the new species do have a small number of jaw teeth. Hutchings and Reid (1990) listed the character of jaw teeth as being present or absent for *Gymnonereis phuketensis*, although the original description by Hylleberg and Nateewathana (1988) states only that they are present (adult specimens, no comments on the juvenile form) but that they can be weakly defined. Where jaw teeth are found in *Gymnonereis tenera* sp. n., however, there are only up to 5 compared to 10 for *Gymnonereis phuketensis*. Additionally, in *Gymnonereis phuketensis* the dorsal cirrophores become “abruptly enlarged” from chaetiger 14 (Hylleberg and Nateewathana 1988) compared to a more gradual enlargement from chaetiger 12 for the new species and the second ventral cirrus is absent from around chaetiger 35 on *Gymnonereis phuketensis* but present throughout on *Gymnonereis tenera* sp. n.

#### Subfamily Nereidinae Blainville, 1818

##### Genus Eunereis

Taxon classificationAnimaliaPhyllodocidaNereididae

Malmgren, 1865

Eunereis Malmgren, 1865: 182–183

###### Type species.

*Nereis longissima* Johnston, 1840

###### Diagnosis

(after Bakken and Wilson 2005). Prostomium with entire anterior margin, one pair of antennae, one pair of biarticulated palps with conical palpostyles, four pairs of tentacular cirri with distinct cirrophores.

Two pairs of eyes. One apodous anterior segment, greater than length of chaetiger 1. Maxillary ring of pharynx without paragnaths. Oral ring, conical paragnaths: Area V, present or absent; VI, present or absent, smooth bars present or absent; VII−VIII, present or absent. Dorsal notopodial ligule present, similar in size or markedly reduced on posterior chaetigers. Prechaetal notopodial lobe present or absent; when present, restricted to a limited number of anterior chaetigers. Acicular process present or absent. Dorsal cirrus basally attached to dorsal notopodial ligule throughout all chaetigers, lacking basal cirrophore. Neuropodial postchaetal lobe absent or present. Notoaciculae absent from chaetigers 1 and 2. Notochaetae: homogomph spinigers present, homogomph falcigers present or absent. Neurochaetae, superior fascicle: homogomph spinigers and heterogomph falcigers present. Neurochaetae, inferior fascicle: heterogomph spinigers and heterogomph falcigers with long blades present.

##### Eunereis patagonica

Taxon classificationAnimaliaPhyllodocidaNereididae

(McIntosh, 1885)

Figure 3

**Figure 3.**
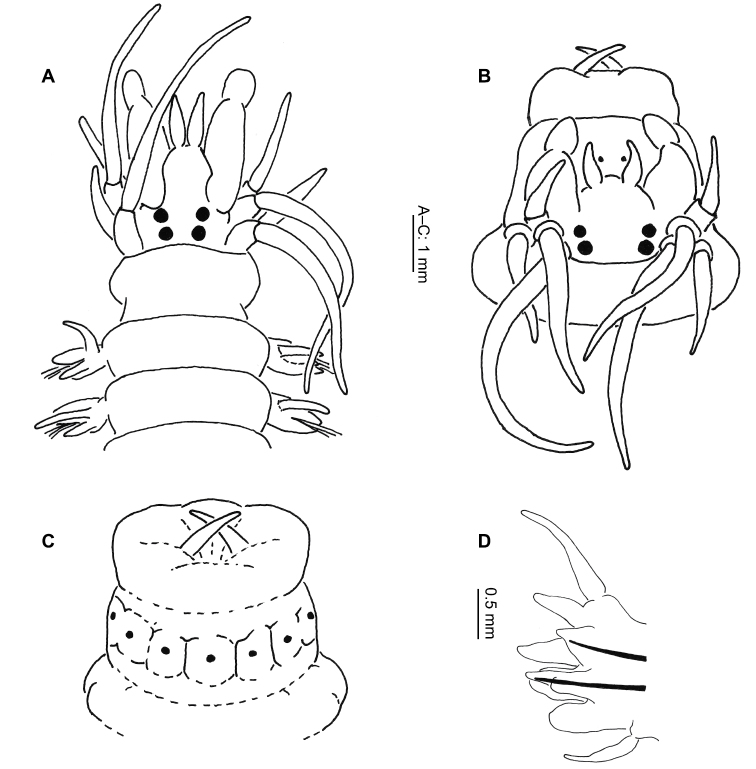
*Eunereis patagonica* (McIntosh, 1885) (after Monro, 1930, as *Nereis (Eunereis) hardyi*): **A** Anterior end, dorsal view **B** Prostomium and proboscis, dorsal view **C** Proboscis, ventral view **D** Parapodium.

**Figure 4.**
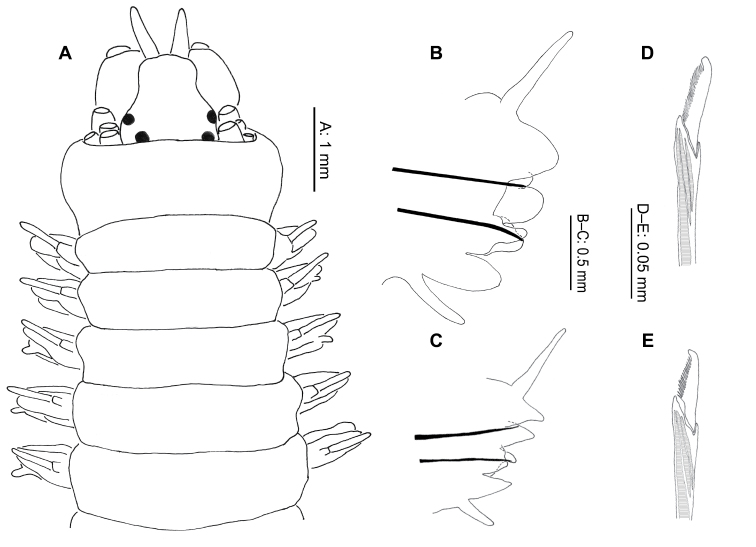
*Neanthes kerguelensis* (McIntosh, 1885) (NMW.Z.2011.039.0127): **A** anterior end (tentacular cirri removed), dorsal view **B** right parapodium, chaetiger 10, posterior view **C** right parapodium, chaetiger 47, posterior view **D** neuropodial heterogomph falciger, chaetiger 10 **E** neuropodial heterogomph falciger, chaetiger 47.

Nereis patagonica McIntosh, 1885: 228–229, Pl. XXXV, figs 13–15, Pl. XVIIA, figs 1–2. — Pratt 1898: 15.
Nereis (Eunereis) hardyi Monro, 1930: 109–111, fig. 39. — Monro 1936: 134–135.
Eunereis patagonica . — Hartman 1953: 29. — Hartman 1964: 97, Pl. XXX, figs 3–4. — Hartman 1967: 62–64, Pl. 15.

###### Material examined.

Strait of Magellan, stn 313 (52°20'S, 067°39'W), sand, 100.6 m, 2 syntypes (NHMUK 1885.12.1.171) 20.01.1876; South America, off Uruguay, stn 1 (33°00'S, 051°10'W), blackish clay, 80 m, 2 specimens (SMNH 37888), 12.12.1901; south of West Falkland, Burdwood Bank, stn 59 (53°45'S, 061°10'W), gravel & stones, 137–150 m, 13 specimens (9–SMNH 37894; 4–SMNH 37902), 12.09.1902; off Falkland Islands, stn WS 86 (53°53'30"S, 060°34'30"W), 6 syntypes *Nereis (Eunereis) hardyi* (NHMUK 1930.10.8.841–844), 03.04.1927; Strait of Magellan, stn WS 834 (52°57'45"S, 068°08'15"W), 4 specimens *Nereis (Eunereis) hardyi* (NHMUK 1936.2.8.1463–1476), 02.02.1932.

###### Description.

Length up to 130 mm, width to 5 mm (excluding parapodia) for up to 85 chaetigers. Eyes present (Fig. 3A–B). Tentacular cirri reaching to chaetiger 6–8 (postero-dorsal pair). Paragnaths absent from maxillary ring; arranged on oral ring as follows (Fig. 3B–C): Area V =1–2; Area VI = 0; Areas VII–VIII = 7–8 in a row. Jaws dark, 5–10 teeth.

Dorsal cirrus longer than notopodia throughout (Fig. 3D), becoming more pronounced posteriorly. Anterior notopodia with dorsal and median ligules conical, median slightly more stout than dorsal. Small, notopodial prechaetal lobe present in anterior chaetigers.

Neuropodia with postchaetal lobe and ventral ligule equal-sized anteriorly; postchaetal lobe conical, reducing in size posteriorly, ventral ligule rounded in the anterior, conical posteriorly.

Notopodia with homogomph spinigers throughout, falcigers absent. Neuropodia with homogomph spinigers and heterogomph falcigers in superior fascicle, inferior fascicle with heterogomph spiniger and falcigers.

Species builds tough-walled tubes coated in sand grains and other coarse particles.

###### Remarks.

The above description is based on McIntosh (1885), Monro (1930, 1936 as *Nereis hardyi*), and Hartman (1953, 1967). However, there is some discrepancy between the original descriptions that can now be clarified following examination of the specimens from those accounts. All three authors agree that paragnaths are absent in Areas I and II and that Area VII–VIII has a single row of 7–8. In Area III, paragnaths are absent on the specimens of both Monro (1930, 1936) and Hartman (1953, 1967), however there is a single conical paragnath present on McIntosh’s specimen. Paragnaths are absent from Area IV on specimens of McIntosh and Monro as well as Hartman’s (1953) Falkland Island specimens (SMNH 37894, 37902) but 3 conical paragnaths are present on her Uruguay specimens (SMNH 37888). The paragnaths described for Area V in Monro (1930, 1936) are present in identical form on Hartman’s (1953) Falkland Island specimens, although she placed them in Area VI in her description, however her Uruguay specimens from the same survey have 3 conical paragnaths in the same position. A single paragnath is present in Area V of McIntosh’s specimen and it is assumed that the second paragnath has been lost or is absent through aberration as McIntosh states in his description that the single paragnath is “nearly, but not quite median”.

It is clear that Hartman’s (1953) Uruguay specimens are a different, currently unidentified species but that her Falkland Island specimens are identical to those of Monro (1930, 1936). Hartman’s 1967 description also agrees with Monro’s and the modified description above reflects these specimens. The presence of the single paragnath in Area III of McIntosh’s specimen requires further investigation as to whether this means that the species needs future re-assessment, as this would place the species in a different genus. Unfortunately, the specimen is in poor condition and a second, smaller specimen from the same location is even worse. However, a comparison of parapodia and what chaetae are available (the vast majority are broken on the McIntosh specimens) show them to be comparable. The paragnath arrangement above is therefore based on the specimens of Monro (1930, 1936) and Hartman (1953, 1967) that were actually collected from the Falkland Islands. The designation of McIntosh’s specimen requires further investigation although as it only deviates from the others in the presence of that single paragnath in Area III it is possible it is aberrant.

*Eunereis patagonica* was first recorded from the Falkland Islands by Pratt (1898) from samples that were probably from intertidal or shallow water samples, however no actual habitat, depth or locality details were given. The only other records from the Falkland Islands are those of Monro (as *Nereis hardyi*: 1930, 1936) and Hartman (1953, 1967) from offshore (106–150 m) samples, as well as an even deeper record at 1879–1886 m by Hartman (1967).

Outside of the region, the species was recorded by Hartman (1967) from 31 m (Cape Horn) to 300 m (South Shetland Islands) together with an additional record of a pelagic epitoke from the Pacific Antarctic Ridge at 3660 m considered to have been carried beyond its viable range.

The species is here believed unlikely to be found intertidally around the Falkland Islands but with potential to be found in the region’s nearshore (< 30 m) waters; Pratt’s 1898 record (if accurate), likely being from this region.

###### Habitat.

Sand, shell, stones; 31–1886 m (?3660 m)

###### Distribution.

Tierra del Fuego, Strait of Magellan, Cape Horn, Falkland Islands, South Shetland Islands, South Orkney Islands, ?Pacific Antarctic Ridge

##### Genus Neanthes

Taxon classificationAnimaliaPhyllodocidaNereididae

Kinberg, 1865

Neanthes Kinberg, 1865: 171

###### Includes.

*Nectoneanthes* Wilson, 1988: 5.

###### Type species.

*Neanthes vaalii* Kinberg, 1865, by original designation

###### Diagnosis

(after Bakken and Wilson 2005). Prostomium with entire anterior margin, one pair of antennae, one pair of biarticulated palps with conical palpostyles, four pairs of tentacular cirri with distinct cirrophores. Eyes present or absent. One apodous anterior segment, greater than length of chaetiger 1. Maxillary ring of pharynx, conical paragnaths: Areas I−IV, present or absent; IV, smooth bar-like paragnaths present or absent. Oral ring, conical paragnaths: Areas V and VI present as distinct groups or not separated; V−VIII, present or absent. Dorsal notopodial ligule present, similar in size on anterior and posterior chaetigers or markedly reduced on posterior chaetigers. Prechaetal notopodial lobe present or absent, smaller than dorsal notopodial ligule on anterior chaetigers, usually reduced or absent posteriorly, present throughout all chaetigers or restricted to a limited number of anterior chaetigers. Acicular process present or absent; present on anterior chaetigers, reducing in size posteriorly. Dorsal cirrus basally or mid-dorsally to subterminally attached to dorsal notopodial ligule on posterior chaetigers, lacking basal cirrophore. Neuropodial postchaetal lobe absent or present. Notoaciculae absent from chaetigers 1 and 2. Notochaetae: homogomph spinigers. Neurochaetae, superior fascicle: heterogomph spinigers present or absent, homogomph spinigers present, heterogomph falcigers on anterior chaetigers present, on posterior chaetigers present or absent. Neurochaetae, inferior fascicle: heterogomph spinigers present or absent, homogomph spinigers present or absent, heterogomph falcigers present.

##### Neanthes kerguelensis

Taxon classificationAnimaliaPhyllodocidaNereididae

(McIntosh, 1885)

Figures 4 , 9C–D

Nereis kerguelensis McIntosh, 1885: 225–227, Pl. XXXV, figs 10–12, Pl. XVIA, figs 17–18. — Augener 1924: 330–333.
Neanthes kerguelensis . — Hartman 1954: 30. — Hartmann-Schröder 1962: 394–395. — Hartman 1967: 64. — Hutchings and Turvey 1982: 113. — Wilson 1984: 216–218. — Bakken and Wilson 2005: 528.

###### Material examined.

East Falkland: Stanley foreshore, stn 1a (51°41.454'S, 057°51.870'W), under rocks in coarse sand, midshore, 3 specimens (NMW.Z.2011.039.0120), 15.11.2011; Stanley foreshore, stn 1b (51°41.459'S, 057°51.840'W), under rocks in coarse sand, midshore, 9 specimens (NMW.Z.2011.039.121), 15.1.2011; Stanley foreshore, stn 1c (51°41.459'S, 057°51.823'W), under rocks in coarse sand, low shore, 3 specimens (NMW.Z.2011.039.0122), 15.1.2011; The Canache, east of Stanley, stn 2c (51°41.716'S, 057°47.107'W), under rocks in gravel & coarse sand, mid-low shore, 6 specimens (NMW.Z.2011.039.0123), 16.1.2011; Hookers Point, stn 4 (51°41.994'S, 057°46.747'W), in & under pink encrusting algae, low shore, 3 specimens (NMW.Z.2011.039.0124), 15.1.2011; Hookers Point, stn 6b, (51°41.994'S, 057°46.747'W), algal holdfast scraping, low shore, 1 specimen (NMW.Z.2011.039.0125), 21.11.2011; Sea Lion Island: East Loafers Bay, stn 20a (52°26.306'S, 059°06.229'W), in & under pink encrusting algae, mid-low shore, 4 specimens (NMW.Z.2011.039.0126), 28.11.2011; East Falkland: west Stanley, stn 21 (51°41.402'S, 057°52.580'W), under small stones in coarse sand & gravel, 6 specimens (NMW.Z.2011.039.0127–0128), 01.12.2011; Egg Harbour, Shag Rookery Point, stn 27 (51°49.345'S, 059°26.719'W), under rocks in soft silty sand, 6 m, 2 specimens (NMW.Z.2011.039.0129), 03.12.2011; Kelp Harbour, stn 29a (51°47.715'S, 059°18.400'W), coralline coarse sand, mid-low shore, 15 specimens (NMW.Z.2011.039.0136), 04.12.2011; Stanley marina, stn 32 (51°41.600'S, 057°48.073'W), *Macrocystis* holdfast, 30 cm, 2 specimens (NMW.Z.2011.039.0132), 05.12.2011; Sand Bay, Port Harriet, stn 34f (51°44.130'S, 058°00.550'W), under rocks within mussel bed, midshore, 7 specimens (NMW.Z.2011.039.0130), 08.12.2011; Teal Creek, east of Darwin, stn 35d (51°49.248'S, 058°55.561'W), under rocks in sand, midshore, 4 specimens (NMW.Z.2011.039.0131), 09.12.2011; Cape Bougainville, stn 38b (51°18.727'S, 058°27.607'W), under rocks in gravel in rock pool, mid-low shore, 1 specimen (NMW.Z.2012.082.0019), 13.01.2013; North Arm, stn 48a (52°07.768'S, 059°22.131'W), mussel bed over silty coarse sand, midshore, 13 specimens (NMW.Z.2013.082.0020), 22.01.2013; West Falkland: Moonlight Bay, Port Stephens, stn 51c (52°06.232'S, 060°50.368'W), in crevices, midshore, 10 specimens (NMW.Z.2012.082.0021), 26.01.2013; The Creek, Hill Cove, stn 56d (51°30.061'S, 060°07.618'W), under algae-covered rocks in fine sand, midshore, 4 specimens (NMW.Z.2012.082.0022), 31.01.2013; Shallow Bay, stn 57e (51°30.032'S, 060°07.726'W), in crevices & under stones, low shore, 3 specimens (NMW.Z.2012.082.0023), 01.02.2013.

###### Description.

Ninety-six entire specimens examined: length 5.9–61.3 mm, width 0.7–3.3 mm (excluding parapodia, measured at 8^th^ chaetiger) for 29–70 chaetigers.

Colour pale cream in alcohol, some with dark brown, uniform shading remaining over anterior chaetigers.

Body depressed dorso-ventrally, of mostly uniform width, tapering in last few chaetigers. Prostomium longer than broad (Fig. 4A), antennae and palps about equal in length, with antennae 1/4 width of palpophores. Palpostyles very short, 1/5 length of palpophores. Four pairs tentacular cirri, postero-dorsal pair extending 2–7 chaetigers, usually 2–3. Two pairs small, equal-sized, black eyes, anterior pair more laterally placed.

Pharynx with conical paragnaths (Fig. 9C, D), variable in size, sometimes faint, not easily lost. Paragnaths arranged as follows: I = 1 (absent or too small to see in specimens of less than 45 chaetigers); II = 1–8; III = 1–9; IV = 6–17; V = 0; VI = 1 (2 on one specimen only); VII–VIII = 3–8. Jaws dark brown to black, 7–10 teeth.

Notopodia with dorsal and median ligule throughout. Of almost equal size, globular anteriorly (Fig. 4B), dorsal ligule becoming conical, median ligule becoming digitiform, in median chaetigers. Notopodial prechaetal lobe present from chaetigers 5–6 (Fig. 4B), increasingly fused to median ligule, absent posteriorly, difficult to determine more precisely due to the very gradual fusion, generally obvious for at least 10 chaetigers.

Dorsal cirrus 1–1.5 times length of dorsal ligule anteriorly (Fig. 4B), increasing to 2–2.5 times length posteriorly (Fig. 4C).

Neuropodia with postchaetal lobe and ventral ligule throughout; postchaetal lobe rounded anteriorly, reduced in size and digitiform posteriorly, ventral ligule globular anteriorly, conical posteriorly (Fig. 4B, C). Ventral cirrus approximately 3/4 length of ventral ligule, becoming equal in length posteriorly (Fig. 4B, C).

Parapodia biramous from chaetiger 3, sub-biramous on chaetigers 1–2. Notochaetae homogomph spinigers only. Neurochaetae with homogomph spinigers and heterogomph falcigers (Fig. 4D, E) in both superior and inferior (from 3) fascicles throughout. No heterogomph spinigers found.

Pygidium terminal; 2 long, tapering anal cirri inserted ventrally.

###### Remarks.

In a detailed study of Australian and sub-Antarctic specimens of *Neanthes kerguelensis*, Wilson (1984) described a wide variation in the numbers of paragnaths in Areas II, III and IV. This, combined with the apparent widespread occurrence across both hemispheres and from intertidal to 5000 m depths, would suggest that records of this species may, in fact, represent a species complex. Greater investigation in each area is required to properly resolve this.

The variation in paragnath numbers exhibited by the Falkland Islands specimens is within the boundaries of that described by Wilson (1984), although it falls consistently at the lower end of those ranges. In addition, the majority of specimens had tentacular cirri that extended only to chaetigers 2–4 (Wilson 1984: 4–8 chaetigers) although some did extend up to chaetiger 7, and the neuropodial postchaetal lobe was present throughout the body as opposed to only the anterior 20–30 chaetigers (Wilson 1984).

There are currently no published genetic sequences for *Neanthes kerguelensis*. However, a comparison of some of these different populations using molecular techniques may help resolve these discrepancies.

###### Habitat.

Wilson (1984) describes the habitat as “associated with fouling communities, intertidal in rocks and sand on sheltered and exposed coasts, soft bottom benthos to 115 m deep”. Previous records from the Falkland Islands exist from intertidal to 197 m depth and from this survey from intertidal to 20 m depth in almost every habitat sampled (including algal holdfasts, epifaunal turf, coarse sand, gravel and under rocks), except for mud and fine-medium clean sands.

###### Distribution.

Recorded widely across the southern hemisphere including Australia, New Zealand, Tasmania, Fiji, Taiwan, Antarctic Peninsula, sub-Antarctic Islands (incl. Kerguelen, Macquarie, South Shetlands, South Orkneys), Chile and the Falkland Islands. Previous records from the Falkland Islands exist from Pratt (1898), Fauvel (1916), Ramsay (1914), Monro (1930) and Hartman (1953) and the species was recorded from almost every location sampled during this survey.

*Neanthes kerguelensis* is also recorded from the Northern hemisphere from the Mediterranean and Azores (von Marenzeller 1902) and the UK (Comely 1973). The latter record, however, is discounted as the author describes his specimen as having 6–7 paragnaths in Area VI which would not identify it as this species.

##### Genus Nereis

Taxon classificationAnimaliaPhyllodocidaNereididae

Linnaeus, 1758

Nereis Linnaeus, 1758: 654.

###### Type species.

*Nereis pelagica* Linnaeus, 1758 (by original designation)

###### Diagnosis

(after Bakken and Wilson 2005). Prostomium with entire anterior margin, one pair of antennae, one pair of biarticulated palps with conical palpostyles, four pairs of tentacular cirri with distinct cirrophores. Eyes present or absent. One apodous anterior segment, greater than length of chaetiger 1. Maxillary ring of pharynx, conical paragnaths: Areas I−III, present or absent; IV, present, smooth bar-like paragnaths present or absent. Oral ring: conical paragnaths present or absent. Dorsal notopodial ligule similar in size in anterior and posterior chaetigers or markedly reduced on posterior chaetigers. Prechaetal notopodial lobe present or absent, smaller than dorsal notopodial ligule on anterior chaetigers, usually reduced or absent posteriorly. Dorsal cirrus basally attached to dorsal notopodial ligule throughout all chaetigers, lacking basal cirrophore. Neuropodial postchaetal lobe absent. Notoaciculae absent from chaetigers 1 and 2. Notochaetae: homogomph spinigers, homogomph falcigers present. Neurochaetae, superior fascicle: homogomph spinigers present, heterogomph falcigers on anterior chaetigers present or absent, on posterior chaetigers present. Neurochaetae, inferior fascicle: heterogomph spinigers present or absent, heterogomph falcigers present or absent.

##### Nereis eugeniae

Taxon classificationAnimaliaPhyllodocidaNereididae

(Kinberg, 1865)

Figure 5

Nicon eugeniae Kinberg, 1865: 178.
Nereis eugeniae . — Ehlers 1897: 67–70, Pl. IV, figs 94–105. — Ehlers 1901: 105, Pl. XII, figs 18–22. — Ramsay 1914: 43. — Monro 1930: 104. — Hartman 1964: 100–101, Pl. XXX, figs 9–10. — Hartman 1967: 65.

###### Description.

**Figure 5.**
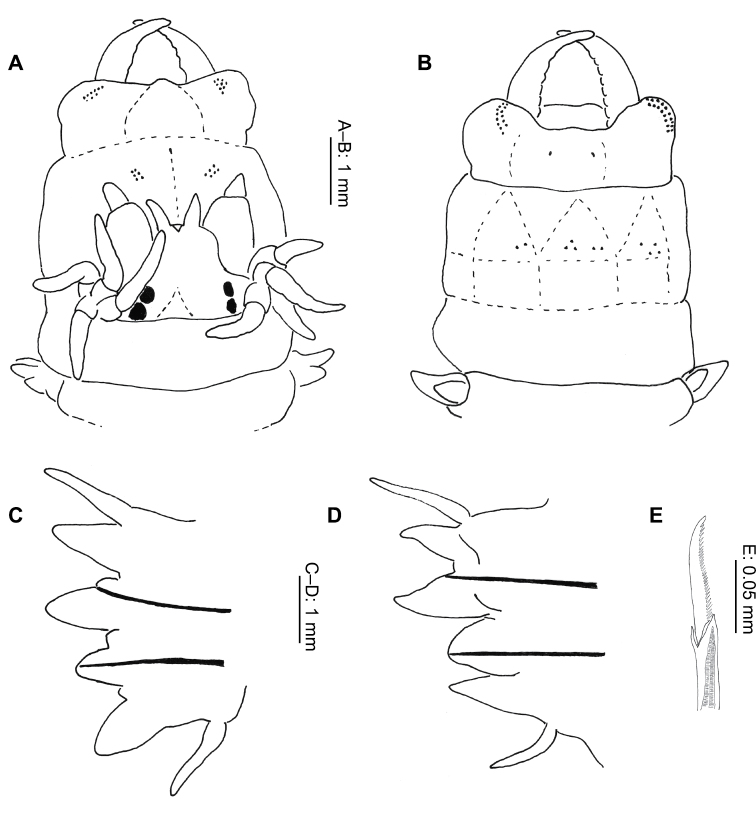
*Nereis eugeniae* Kinberg, 1865 (after Ehlers 1897): **A** anterior end, dorsal view **B** chaetiger 12 **C** chaetiger 37 **D** neuropodial heterogomph spiniger, posterior chaetiger **E** neuropodial heterogomph falciger, posterior chaetiger.

Length up to 170 mm, width up to 3 mm including parapodia for up to 125 chaetigers. Eyes absent or present. Paragnaths arranged as follows (Fig. 5A, B): Area I = 0; Area II = small group (up to 11); Area III = absent or sparse, irregular row (2–6); Area IV = absent or group (0–18); Area V = 0–1; Area VI = small group (3–6); Areas VII–VIII = sparse, irregular row (0–11). Jaws dark, 5–7 teeth.

Dorsal cirrus longer than notopodia throughout, becoming more pronounced posteriorly. Anterior notopodia (Fig. 5C) with dorsal and median ligules equal in size, dorsal ligule reducing in size posteriorly. Small notopodial prechaetal lobe present in anterior chaetigers.

Neuropodia with postchaetal lobe and ventral ligule conical; postchaetal lobe shorter than notopodial ligules and ventral ligule in anterior chaetigers, becoming more equal in size posteriorly (Fig. 5D).

Anterior notopodia with homogomph spinigers only, 2–3 homogomph falcigers present from median chaetigers on. Neuropodia with homogomph spinigers and heterogomph falcigers in superior fascicle, inferior fascicle with heterogomph spinigers and falcigers (Fig. 5E).

###### Remarks.

The above description is an amalgamation of the information provided by Ehlers (1897), Monro (1930) and Hartman (1964, 1967), although of these, only Monro published on specimens from the Falkland Islands. The type locality for the species is the Strait of Magellan, but Kinberg (1865) gave little detail about the animal itself. The species was later comprehensively re-described and drawn by Ehlers (1897). Descriptions by different authors are quite variable, particularly regarding the paragnaths arrangements. Ramsay (1914) gave no details about his specimens except to say that they “agreed in all respects” with Ehlers’ description whereas Monro (1930) noted that, in contrast to Ehlers’ description, the paragnaths of Areas VII–VIII “form a single very sparse irregular row and in a number of the larger examples they appear to be altogether absent”

*Nereis eugeniae* was not collected by this survey, however it has been recorded from several offshore locations around the islands from 1–115 m (Ramsay 1914; Monro 1930) and Monro (1930) described the species as being “common off the Falkland Islands”. There are no intertidal records for the area, however *Nereis eugeniae* has been recorded intertidally from Chile (Ehlers 1901; Hartman 1967). Although not recorded here, the species is known to be present in shallow water around the islands and could potentially be found intertidally also.

###### Habitat.

Sand, shell, stones, cobbles; intertidal–156 m.

###### Distribution.

Strait of Magellan, Chile, Falkland Islands, Kerguelen Islands, Patagonia.

##### Genus Perinereis

Taxon classificationAnimaliaPhyllodocidaNereididae

Kinberg, 1865

Perinereis Kinberg, 1865: 175–176. — Hutchings et al. 1991: 245.

###### Includes.

*Arete* Kinberg, 1865; *Gnatholycastis* Ehlers, 1920.

###### Type species.

*Perinereis novaehollandiae* Kinberg, 1865; by subsequent designation (Hartman 1948)

###### Diagnosis

(after Bakken and Wilson 2005, emended). Prostomium with entire anterior margin, one pair of antennae, one pair of biarticulated palps with conical palpostyles, four pairs of tentacular cirri with distinct cirrophores. Two pairs of eyes. One apodous anterior segment, greater than length of chaetiger 1. Maxillary ring of pharynx, conical paragnaths: Area I, present or absent; II, present or absent; III, present; IV, present or absent, smooth bar-like paragnaths present or absent. Oral ring, conical paragnaths: Area V, present or absent; VI, present or absent, *smooth or shield-shaped* bars present; VII−VIII, present. Dorsal notopodial ligule similar in size in anterior and posterior chaetigers, or markedly elongate on posterior chaetigers. Prechaetal notopodial lobe present or absent, smaller than dorsal notopodial ligule on anterior chaetigers, usually reduced or absent posteriorly. Dorsal cirrus basally or mid-dorsally to subterminally attached to dorsal notopodial ligule on posterior chaetigers, lacking basal cirrophore. Neuropodial postchaetal lobe absent or present. Notoaciculae absent from chaetigers 1 and 2. Notochaetae: homogomph spinigers. Neurochaetae, superior fascicle: homogomph spinigers and heterogomph falcigers present. Neurochaetae, inferior fascicle: heterogomph spinigers present or absent, heterogomph falcigers present.

##### Perinereis atlantica

Taxon classificationAnimaliaPhyllodocidaNereididae

(McIntosh, 1885) comb. n.

Figure 6

Nereis atlantica McIntosh, 1885: 219–221, Pl. XXXV, figs 1–3, Pl. XVIa, figs 10–11. — Pratt 1898: 16.
Nereis atlantica ? . — Hartman 1964: 99, Pl. XXX, figs 7–8.

###### Material examined.

St Vincent, Cape Verde Islands (NHMUK.1885.12.1.161), holotype, July 1873.

###### Description.

**Figure 6.**
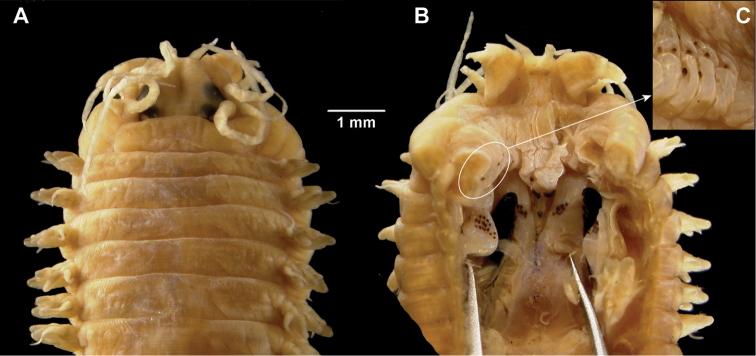
*Perinereis atlantica* (McIntosh, 1885) (NHMUK.1885.12.1.161): **A** anterior end, dorsal view **B** anterior end, ventral view **C** enlarged view of partial Area VII–VIII of proboscis.

Examination of the holotype (Fig. 6A–C), shows the description and illustrations by McIntosh to be quite accurate. The only refinements are as follows:

Body dorso-ventrally depressed, mostly of uniform width, gradually tapering in last 20–30 chaetigers to pygidium.

Paragnaths arranged as follows, all conical except for Area VI (Fig. 6B–C): Area I = 1 large, Area II = 6–8, Area III = 8, Area IV = 15–16 arranged in 3–4 rows, Area V = 1 small, Area VI = 1 shield-shaped bar with rounded apex, Area VII–VIII = 3 rows with 6 (distal row), 9 (middle row) & 4 (proximal row) evenly-spaced cones, middle and proximal cones more flattened and blunt than those of the distal row. Jaws robust, dark brown with 4 teeth (Fig. 6B).

Dorsal ligule expanded posteriorly to a greater extent than figured by McIntosh but not as much as *Perinereis falklandica*.

Notochaetae all homogomph spinigers, neurochaetae homogomph and heterogomph spinigers and heterogomph falcigers (from observations of a limited number of chaetae, most broken so distribution between inferior and superior fascicles unknown). Falciger tips become shorter posteriorly but otherwise do not change in form along the body.

Pygidium terminal; 3 long, thin anal cirri of equivalent length to last 11 chaetigers (1 cirrus apparently lost as McIntosh’s original description states 4 anal cirri, 2 each side of anus). Pygidium and last 3 chaetigers with appearance of regeneration.

###### Remarks.

This species was described from a single specimen collected at Cape Verde Islands in the southeast Atlantic. McIntosh (1885) noted that the species appeared most closely related to *Perinereis*, however, the large, bar-shaped paragnaths characteristic of that genus were present in Area V not VI, Area VI being empty. He related the species most closely to *Nereis floridana* Ehlers, 1868, now *Perinereis floridana* (Ehlers, 1868) and would most likely have also placed *Nereis atlantica* into *Perinereis* if *Perinereis floridana* had already been placed there. The lack of notopodial falcigers would also now place it outside of *Nereis*. It is believed that the specimen is aberrant, with the large bars of Area VI here situated much closer together than would normally be expected and appearing to be in Area V instead (the aberration appears to be more than just an artifact of contraction). The additional cone behind one of the bars could be skewed out of position from Area V or may be an aberrant additional cone in Area VI (it is here assumed to be out of position from Area V due to an aberration). An additional sign of possible aberration is that of the 4 (currently 3) anal cirri on a regenerating pygidium. More material will be required from the type locality to determine the true form and validity of the species. Until then, *Nereis atlantica* is transferred to *Perinereis* based on the large, bar-shaped paragnaths and the lack of notopodial falcigers.

Since its description, the only other record of the species has been by Pratt (1898) from Hill Cove on West Falkland (southwest Atlantic) although Hartman (1964) cast doubt on the validity of this record due to the distance from its original locality. Unfortunately, both McIntosh and Pratt gave only general locality details for their specimens and no details of habitat or depth. However, as Pratt’s specimens generally came from shore or shallow water samples it is assumed that her *Nereis atlantica* were either intertidal or nearshore. Attempts to locate the specimens at Manchester (where she worked), Cambridge (where the other specimens she published on were loaned from) and the Natural History Museum, London have proved fruitless. The record from the Falkland Islands is therefore also considered doubtful in this paper. It is possible that, with *Perinereis falklandica* undescribed at that time and, as a student working on Bryozoa and not Annelida, Pratt mistakenly identified *Perinereis falklandica* as *Nereis atlantica*. Unfortunately, without the specimens no confirmation of this is possible. Certainly, aside from Pratt’s record, no other specimens like *Perinereis atlantica* have ever been reported from the Falkland Islands.

With the shield-shaped bars now re-described into Area VI, the species would fall into ‘Group 1A’ of Hutchings et al. (1991) along with *Perinereis floridana*: *Perinereis* species with 1 bar in Area VI and dorsal notopodial lobe not greatly expanded.

###### Habitat.

Unknown.

###### Distribution.

Cape Verde Islands, ?Falkland Islands.

##### Perinereis falklandica

Taxon classificationAnimaliaPhyllodocidaNereididae

(Ramsay, 1914)

Figure 7 , 9E–F

Nereis (Perinereis) falklandica Ramsay, 1914: 44–46, pl. 3, figs 3–10.
Perinereis falklandica . — Fauvel 1941: 280–281. — Hartman 1953: 29. — Day, 1954: 18. — Wesenberg-Lund 1962: 80–83, figs 30–31. — Hartmann-Schröder 1962: 410–411. — Hartmann-Schröder 1965: 298–299. — Rozbaczylo and Castilla 1973: 218–220, fig 2. — Rozbaczylo and Bolados 1980: 214–216. — Sampertegui et al. 2013: 30, fig. 1.

###### Material examined.

East Falkland: The Canache, east of Stanley, stn 2c (51°41.716'S, 057°47.107'W), under rocks in gravel & coarse sand, mid-low shore, 9 specimens (NMW.Z.2011.039.0108–0109), 16.1.2011; Hookers Point, stn 6a, (51°41.994'S, 057°46.747'W), under pink encrusting algae, low shore, 3 specimens (NMW.Z.2011.039.0110), 21.11.2011; Hookers Point, stn 6c, (51°41.994'S, 057°46.747'W), under pink encrusting algae, low shore, 3 specimens (NMW.Z.2011.039.0111), 21.11.2011; Hookers Point, stn 6d, (51°41.994'S, 057°46.747'W), in silty gravel washings from rock pool, low shore, 1 specimen (NMW.Z.2011.039.0112), 21.11.2011; Egg Harbour, stn 25 (51°50.353'S, 059°27.351'W), rocks & mussel bed in silty coarse sand, mid-low tide, 12 specimens (NMW.Z.2011.039.0114), 03.12.2011; Sea Lion Island: East Loafers Bay, stn 20a (52°26.306'S, 059°06.229'W), in & under pink encrusting algae, mid-low shore, 1 specimen (NMW.Z.2011.039.0113), 28.11.2011; Saunders Island: The Neck, stn 42d (51°18.485'S, 060°14.504'W), under stones on rock ledges, midshore, 3 specimens (NMW.Z.2012.082.0011), 17.01.2013; West Falkland: Shallow Bay, stn 57b (51°30.032'S, 060°07.726'W), in crevices & under stones, high-mid shore, 2 specimens (NMW.Z.2012.082.0012), 01.02.2013; Shallow Bay, stn 57c (51°30.032'S, 060°07.726'W), in crevices & under stones, low shore, 5 specimens (NMW.Z.2012.082.0013), 01.02.2013.

###### Description.

Thirty-nine entire specimens examined; length 19.5–73.6 mm, width (excluding parapodia) 1.5–4.3 mm for 65–89 chaetigers.

Colour in alcohol, dark brown body with pale parapodia, colour becoming paler more posteriorly, variably according to specimen. Head very dark green/brown with pale median line (Fig. 9E). Live colour green-brown with pale markings as described in alcohol.

**Figure 7.**
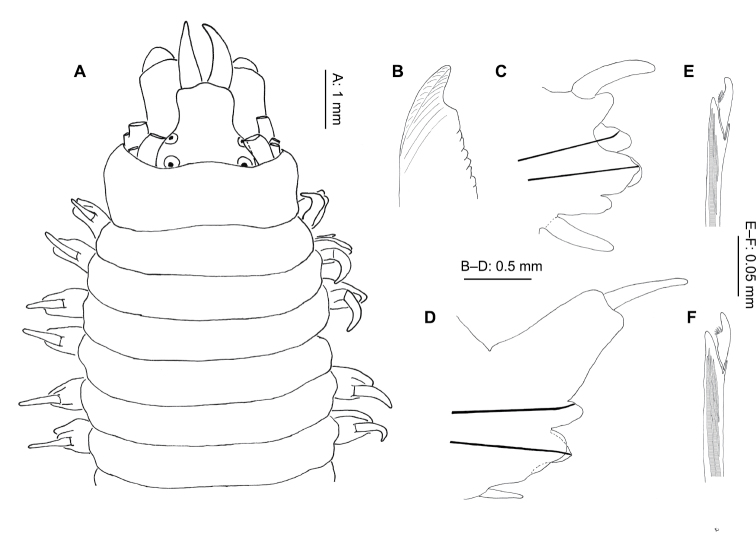
*Perinereis falklandica* Ramsay, 1914 (NMW.Z.2011.039.0108): **A** anterior end (tentacular cirri & right chaetiger 4 removed), dorsal view **B** jaw **C** right parapodium, chaetiger 4, posterior view **D** right parapodium, chaetiger 71, posterior view **E** neuropodial heterogomph falciger, chaetiger 4 **F** neuropodial heterogomph falciger, chaetiger 71.

Body dorso-ventrally depressed, uniform width for most of length, tapering slightly over last few chaetigers. Head with prostomium longer than broad (Fig. 7A), antennae short, stout, 2/3 length of broad palps. Four pairs short, tentacular cirri, pale with dark cirrophores, reaching to chaetiger 2–4. Two pairs small, black eyes, equal size, anterior pair more laterally placed (Fig. 7A). Eyes difficult to discern once preserved due to dark prostomial colour, particularly anterior pair.

Proboscis with conical (except for Area VI) paragnaths (Fig. 9E, F), variable in size and number, arranged as follows: Area I = 1 large, central surrounded by triangle of 32–150 small, faint, blunter cones; II = broad triangle of large and small cones, 9–28 each side; III = oval patch of 11–20 medium-sized cones; IV = curved lines of 23–40 small–large cones; V = 1 large, blunt cone (1 aberrant specimen with 1 large & over 20 small cones); VI = 1 large, shield-shaped bar with pointed apex; VII–VIII = 2–3 single, large cones laterally, almost reaching Area VI, becoming a broad swath ventrally of 110–300 large and small blunt cones. Jaws dark black/brown with 5–10 teeth and large distal fang (Fig. 7B).

Anterior notopodia with dorsal and median ligules rounded anteriorly (Fig. 7C), becoming conical in median chaetigers; dorsal ligule swollen and elongated from around chaetiger 50 (Fig. 7D).

Neuropodia with conical postchaetal lobe and ventral ligule anteriorly, ventral ligule smaller, almost absent posteriorly.

Notochaetae homogomph spinigers throughout, figured specimen with 13 on chaetiger 4, reducing posteriorly to 6 on chaetiger 71 (of 89). Neurochaetae with homogomph spinigers in superior fascicle only (chaetiger 4: 5, chaetiger 71: 6), heterogomph falcigers present in both superior (chaetiger 4: 5; chaetiger 71: 3) and inferior (chaetiger 4: 15, chaetiger 71: 8) fascicles throughout, little change in form along body (Fig. 7E, F). Inferior fascicle with falcigers arranged in a C-shape on anterior chaetigers, thereafter in a transverse line.

Pygidium terminal; two short, anal cirri inserted ventrally.

###### Habitat.

In this study, all specimens were from intertidal, mid-low shore locations, in hard substrates such as coarse sand/gravel, under rocks, in crevices and under pink encrusting algae.

Of the handful of other records in the literature, the species is mostly found intertidally in hard, often exposed habitats. Ramsay (1914) collected his specimens from 15 fathoms (27.4 m), the deepest record of this species.

###### Distribution.

Falkland Islands, Magellan region (Orange Bay), Tristan da Cunha, Chile

###### Remarks.

*Perinereis falklandica* has not been reported very widely in the literature since Ramsay described it from the Falkland Islands in 1914, although it was found to be quite common in coarse, intertidal habitats during this survey. Only one other record exists for the locality, being that of Hartman (1953), from a single intertidal sample at Port Louis. This is undoubtedly due to the fact that, other than Ramsay’s original record, the species has rarely been identified from sublittoral samples and little intertidal work has been undertaken in the Falkland Islands. Outside of the Falkland Islands, with the exception of a single record from Tristan da Cunha (Day, 1954), it is mostly known from the coast of Chile (Fauvel 1941 (Magellan Strait); Wesenberg-Lund 1962; Hartmann-Schröder 1962, 1965; Rozbaczylo and Castilla 1973; Rozbaczylo and Bolados 1980; Sampertegui et al. 2013).

The validity of the species has not been questioned and it is easily distinguishable from other species. Type material was therefore not examined.

Descriptions of the specimens from the different localities are mostly uniform with the only variation being in the number of paragnaths found in Area V of the proboscis. Most authors have reported a single, large cone in this region with the exception of Day (1954; 1–3 cones), Rozbaczylo and Castilla (1973; 1–5 cones) and Sampertegui et al. (2013; 1–3 cones). All of the specimens in the current study exhibited only a single cone with the exception of one aberrant specimen with 1 large and 27 small cones. The latter specimen agrees with the usual description of *Perinereis falklandica* in all other respects and is considered aberrant. The number of paragnaths in Areas I–IV and VII–VIII are highly variable and the range exhibited by the specimens in the current study fall within the larger range reported by Sampertegui et al. (2013).

Hutchings et al. (1991) placed *Perinereis falklandica* into their ’Group 1B’: *Perinereis* species with 1 bar in Area VI and dorsal notopodial lobe greatly expanded on posterior chaetigers.

##### Genus Platynereis

Taxon classificationAnimaliaPhyllodocidaNereididae

Kinberg, 1865

###### Includes.

*Iphinereis* Malmgren, 1865; *Pisenoë* Kinberg, 1865; *Leontis* Malmgren, 1867; *Nectonereis* Verrill, 1873; *Uncinereis* Chamberlin, 1919.

###### Type species.

*Platynereis magalhaensis* Kinberg, 1865, by subsequent designation (Hartman 1948)

###### Diagnosis

(after Read 2007, emended). Proboscis with chitinous paragnaths in form of parallel, *tight* rows of minute *rods* usually present on all areas except I, II and V. Prostomium with 2 antennae, biarticulate palps and 2 pairs of eyes; 4 pairs of tentacular cirri. Peristomial segment apodous and first 2 parapodia sub-biramous. Chaetae include spinigers and falcigers. Homogomph notopodial falcigers usually present, in least in juveniles.

###### Remarks.

The above description is emended with respect to the paragnath terminology introduced by Bakken et al. (2009). However, it should be noted that Bakken et al. (2009) only confirmed the form of paragnaths as tight rows of rods, as opposed to the previously described pectinate bars, for 3 species of *Platynereis* that did not include *Platynereis magalhaensis*. This is now, however, confirmed for *Platynereis magalhaensis* below.

##### Platynereis magalhaensis

Taxon classificationAnimaliaPhyllodocidaNereididae

Kinberg, 1865

Figures 8 , 9G–I

Platynereis magalhaensis Kinberg, 1865: 177. — 1910: 53, Pl. XX, fig. 6. — Pratt 1901: 2. — Fauvel 1916: 434–436, Pl. VIII, figs 21–22. — Monro 1930: 106–107, fig. 37. — Hartman 1948: 60–61.
Platynereis patagonica Kinberg, 1865: 177.
Platynereis antarctica Kinberg, 1865: 177.
Pisenoë maculata Kinberg, 1865: 176.
Nicon loxechini Kinberg, 1865: 178–179.
Nereis antarctica Verrill, 1876.
Nereis eatoni McIntosh, 1876: 320.
Nereis (Platynereis) eatoni McIntosh, 1885: 223–224, Pl. XXXV, figs 5–6.
Nereis magalhaensis . — Ehlers 1897: 63–65, Pl. V, figs 106–107.

###### Material examined.

East Falkland: Stanley foreshore, stn 1c (51°41.459'S, 057°51.823'W), under rocks in coarse sand, low shore, 1 specimen (NMW.Z.2011.039.0145), 15.1.2011; The Canache, east of Stanley, stn 2e (51°41.731'S, 057°47.001'W), medium sand, low shore, 4 specimens (NMW.Z.2011.039.0146), 16.1.2011; Cochon Island: stn 10 (51°36.287'S, 057°47.684'W), under rocks, 9.5 m, 14 specimens (NMW.Z.2011.039.0147–0149), 24.11.2011; stn 11 (51°36.377'S, 057°489'W), under rocks, 9.6 m, 10 specimens (NMW.Z.2011.039.0150), 24.11.2011; stn 13 (51°36.322'S, 057°47.132'W) epifaunal turf scraping, 13.6 m, 3 specimens (NMW.Z.2011.039.0141), 25.11.2011; stn 15a (51°36.449'S, 057°47.150'W), under rocks, 18.0 m, 1 specimen (NMW.Z.2011.039.0151), 26.11.2011; stn 16b (51°36.366'S, 057°47.082'W), epifaunal turf scraping, 12.5 m, 1 specimen (NMW.Z.2011.039.0142), 26.11.2011; Kidney Island: stn 18b (51°37.517'S, 057°45.301'W), fine-medium sand, 4.6 m, 2 specimens (NMW.Z.2011.039.0152), 27.11.2011; East Falkland: west Stanley, stn 21 (51°41.402'S, 057°52.580'W), under small stones in coarse sand & gravel, 2 specimens (NMW.Z.2011.039.0153), 01.12.2011; Egg Harbour, stn 22 (51°47.471'S, 059°24.360'W), under rocks, 13.9 m, 4 specimens (NMW.Z.2011.039.0157), 02.12.2011; Egg Harbour, stn 23 (51°49.477'S, 059°23.926'W), under rocks, 11.6 m, 5 specimens (NMW.Z.2011.039.0143), 03.12.2011; Egg Harbour, Shag Rookery Point, stn 27 (51°49.345'S, 059°26.719'W), under rocks, 6 m, 1 specimen (NMW.Z.2011.039.0154), 03.12.2011; Kelp Harbour, stn 30 (51°47.021'S, 059°19.848'W), under rocks, 9.3 m, 4 specimens (NMW.Z.2011.039.0144), 04.12.2011; Sand Bay, Port Harriet, stn 34f (51°44.130'S, 058°00.550'W), under rocks within mussel bed, midshore, 4 specimens (NMW.Z.2011.039.0155), 08.12.2011; Teal Creek, east of Darwin, stn 35d (51°49.248'S, 058°55.561'W), under rocks in sand, midshore, 1 specimen (NMW.Z.2011.039.0156), 09.12.2011; Race Point Farm, Port San Carlos, stn 37a (51°30.276'S, 059°00.137'W), in crevices, mid-low shore, 3 specimens (NMWZ.2012.082.0041–0042), 12.01.2013; Race Point Farm, Port San Carlos, stn 37b (51°30.277'S, 059°00.080'W), in crevices, low shore, 2 specimen (NMWZ.2012.082.0043), 12.01.2013; Race Point Farm, Port San Carlos, stn 37c (51°30.276'S, 059°00.137'W), under stones, low shore, 1 specimen (NMWZ.2012.082.0044), 12.01.2013; Race Point Farm, Port San Carlos, stn 37d (51°30.276'S, 059°00.137'W), among rocks & gravel in muddy sand, low shore, 1 specimen (NMWZ.2012.082.0045), 12.01.2013; Cape Bougainville, stn 38a (51°18.720'S, 058°27.603'W), in pink encrusting algae in open crevices, low shore, 2 specimens (NMW.Z.2012.082.0047), 13.01.2013; Cape Bougainville, stn 38b (51°18.727'S, 058°27.607'W), under rocks in gravel in rock pool, mid-low shore, 2 specimens (NMW.Z.2012.082.0048), 13.01.2013; Saunders Island: Sealer Cove harbor, stn 44c (51°21.760'S, 060°04.896'W), under rocks in sandy gravel, low shore, 2 specimens (NMW.Z.2012.082.0049); 18.01.2013; Sealer Cove harbor, stn 44d (51°21.760'S, 060°04.896'W), under rocks in sandy gravel, low shore, 3 specimens (NMW.Z.2012.082.0050); 18.01.2013; East Falkland: North Arm, stn 48a (52°07.768'S, 059°22.131'W), mussel bed over silty coarse sand, midshore, 1 specimen (NMW.Z.2013.082.0051), 22.01.2013; North Arm, stn 48b (52°07.829'S, 059°22.079'W), coarse loose sand, mid-low shore, 1 specimen (NMW.Z.2013.082.0052), 22.01.2013; New Haven, stn 49b (51°43.855'S, 059°12.894'W), under rocks in sandy gravel, mid-low shore, 1 specimen (NMW.Z.2012.082.0054), 24.01.2013; West Falkland: Moonlight Bay, Port Stephens, stn 51d (52°06.266'S, 060°50.334'W), in crevices, mid-low shore, 1 specimen (NMW.Z.2012.082.0055), 26.01.2013; Hot Stone Cove Creek, Dunbar, stn 54g (51°22.883'S, 060°30.886'W), associated with large tunicate attached to rock, low shore, 1 specimen (NMW.Z.2012.082.0056), 29.01.2013; Shallow Bay, stn 57c (51°30.032'S, 060°07.726'W), in crevices & under stones, mid shore, 2 specimens (NMW.Z.2012.082.0057), 01.02.2013.

###### Description.

Eighty-three entire specimens, juveniles to adults, were examined: length 1.9–105.1 mm, width 0.27–4.7 mm (excluding parapodia, measured at chaetiger 4–5) for 16–115 chaetigers. Description based on adult specimens only, defined by the absence of notopodial falcigers.

Colour pale in alcohol.

Body shape depressed dorso-ventrally, mostly of uniform width to posterior, then tapering in last few chaetigers.

**Figure 8.**
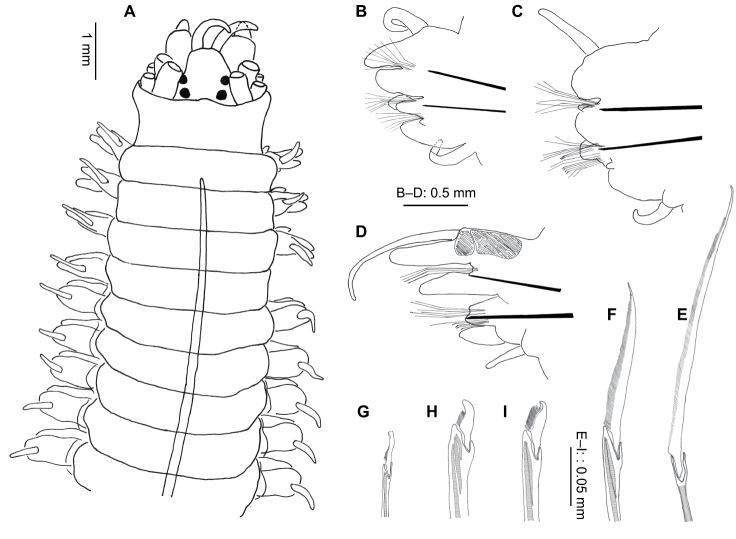
*Platynereis magalhaensis* Kinberg, 1865 (**A–F**, **H–I** NMW.Z.2011.039.0159 **G** NMW.Z.2011.039.0149): **A** anterior end (tentacular cirri & right chaetiger 4 removed), dorsal view **B** right parapodium, chaetiger 4, posterior view **C** right parapodium, chaetiger 10, posterior view **D** right parapodium, chaetiger 71, posterior view; right parapodium, chaetiger 4, posterior view **E** notopodial homogomph spiniger, chaetiger 10 **F** neuropodial heterogomph spiniger, chaetiger 10 **G** juvenile notopodial heterogomph falciger, chaetiger 20 **H** neuropodial heterogomph falciger, chaetiger 10 **I** neuropodial heterogomph falciger, chaetiger 71.

Prostomium longer than broad (Fig. 8A), antennae and palps about equal in length; antennae 1/2–1/3 width of palpophores. Four pairs tentacular cirri, postero-dorsal pair longest, reaching to chaetiger 11–14, rarely 16. Two pairs small, dark brown to black eyes, anterior pair marginally smaller, more laterally placed (Fig. 8A). Mid-dorsal nuchal cushion present, projecting forward slightly on to head from apodous peristomial segment (Fig. 8A). Peristomium approximately one third longer than following segments.

**Figure 9.**
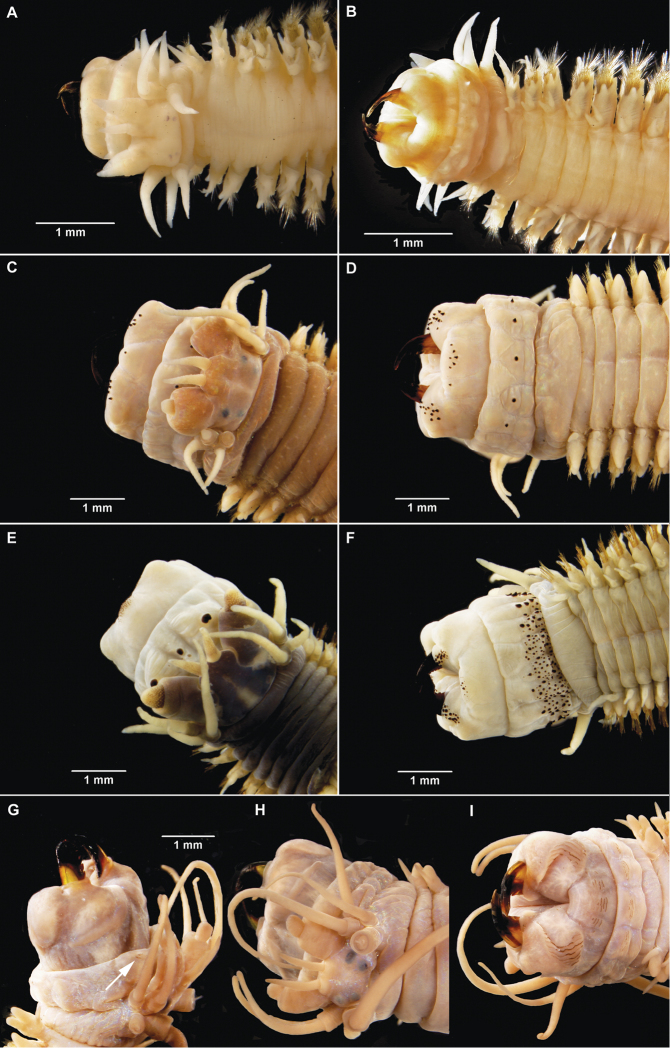
Images of the paragnaths of the species collected. *Gymnonereis tenera* sp. n. (NMW.Z.2011.039.0093): **A** dorsal view **B** ventral view; *Neanthes kerguelensis* (NMW.Z.2011.039.0129) **C** dorsal view **D** ventral view; *Perinereis falklandica* (NMW.Z.2011.039.0113) **E** dorsal view; F. ventral view; *Platynereis magalhaensis* (NMW.Z.2011.039.0158) **G** lateral view (arrow indicating Area VI paragnaths) **H** dorsal view **I** ventral view.

Proboscis with tight lines of rod-like paragnaths in Areas III, IV, VI, VII and VIII, absent in Areas I, II and V. Largest group in area IV with up to 9 long rows, innermost 3–4 rows incomplete. Area III with 3 small groups of up to 4 lines in each. Area VI, the smallest group, often faint, difficult to discern, with up to 3 short lines of rods (Fig. 9G, indicated by arrow). Area VII–VIII with 5 groups of up to 3 curved lines in each (Fig. 9I). Jaws dark brown with up to 12 teeth (Fig. 8G, I).

Parapodia subbiramous on chaetigers 1–2, biramous from chaetiger 3. Parapodial ligules thickened and rounded on chaetigers 5–11, sometimes, to a lesser extent, starting from chaetiger 4 and extending to chaetiger 12, occasionally 13, in larger animals (Fig. 8B, C).

From mid-body dorsal ligule lengthened and glandular (Fig. 8D). Dorsal cirrus longer than dorsal ligule throughout body, minorly so anteriorly, becoming more pronounced and elongate posteriorly (Fig. 8B–D).

Notochaetae homogomph spinigers (Fig. 8E), up to 25–30 per fascicle in mid-body, reduced to around 5 in last few chaetigers. Single heterogomph notopodial falciger, bifid with connecting tendon from tip (Fig. 8G), present in juveniles up to around 60–65 chaetiger stage, absent in adults. First occurrence of notopodial falciger retreats posteriorly as size increases, from around chaetiger 8 (of 16) to chaetiger 62 (of 64).

Neurochaetae homogomph and heterogomph spinigers (Fig. 8F) and heterogomph falcigers (Fig. 8H, I). Superior fascicle spinigers homogomph, up to 8, inferior fascicle spinigers heterogomph, up to 6 (usually 2–3). Falcigers heterogomph, from chaetiger 5 onwards; up to 7 above acicula, up to 17 below; greatest numbers mid-body reducing posteriorly.

Pygidium terminal; two long, thin anal cirri inserted ventrally.

Tube soft, with coarse grains of sand, shell and foraminifera adhered to it.

###### Remarks.

*Platynereis magalhaensis* was the most common nereidid collected by diving with most rocks turned over having tubes attached to the underside. It was also widespread intertidally, again in tubes attached to rocks or algal holdfasts.

The original description of *Platynereis magalhaensis* by Kinberg (1865) was brief with little detail except a general description of the head, and a statement that the tentacular cirri reached to the 15^th^ segment and there were 12 teeth on the jaws. Several authors since then have expanded the description using either newly collected specimens (e.g. Ehlers 1897; Fauvel 1916) or by re-examining Kinberg’s type material (Hartman 1948). The species can be distinguished from most other *Platynereis* species on a combination of the absence of paragnaths in Areas I, II and V and the absence of notopodial falcigers (in adults). However, *Platynereis magalhaensis* remains difficult, if not impossible, to separate morphologically from the *Platynereis australis* ‘group’ — *Platynereis australis* (Schmarda, 1861), *Platynereis karaka* Read, 2007, *Platynereis kau* Read, 2007, *Platynereis mahanga* Read, 2007 — resulting in a conflict of opinion with some authors synonymizing it with *Platynereis australis* while others prefer to keep them separate.

Most recently, a detailed comparison of the *Platynereis australis* group with *Platynereis magalhaensis* was published by Read (2007), following which he concluded that while morphologically inseparable as atokes, as epitokes the species could be differentiated on the basis of characters such as number of pre-natatory segments and male pygidial form and thus *Platynereis magalhaensis* should still be considered a valid species.

Unfortunately, no epitokous forms were among the specimens collected from the Falkland Islands so this aspect cannot be confirmed in this study. However, the few records of epitokes that do exist for this region (Ehlers 1897; Augener 1923; Monro 1930) indicate that the species is likely to be distinct from *Platynereis australis* and Read (2007) additionally stated that records of *Platynereis australis* outside of New Zealand should be re-assessed. The species collected from the Falkland Islands is therefore viewed as being appropriately placed under the name *Platynereis magalhaensis*. However, further study of the epitokous form from the islands is necessary to help clarify the situation.

### Key to intertidal and nearshore Nereididae in the Falkland Islands

**Table:** 

1	Chitinous paragnaths present on pharynx; single ventral cirrus present throughout	2
–	Chitinous paragnaths absent from pharynx; double ventral cirri present	*Gymnonereis tenera* sp. n.
2	Paragnaths present as shield-shaped bars and /or variably-sized cones; chaetiger 5–10 parapodial lobes not noticeably different from lobes on remaining chaetigers	3
–	Paragnaths present as tight rows of rods; chaetigers 5–10 with globular parapodial lobes	*Platynereis magalhaensis* Kinberg, 1865
3	Area VI with paragnaths as cones, shield-shaped bar with rounded apex or absent; posterior notopodial dorsal lobes not noticeably enlarged	4
–	Area VI with 1 large, shield-shaped bar with pointed apex; posterior dorsal notopodial lobes greatly enlarged	*Perinereis falklandica* Ramsay, 1914
4	Falcigers absent in notopodia	5
–	Falcigers present in at least some notopodia	6
5	Paragnaths absent on maxillary ring and Area VI; ventral fascicle of neuropodia includes heterogomph spinigers	*Eunereis patagonica* (McIntosh, 1885)
–	Paragnaths present on maxillary ring and Area VI; all spinigers homogomph, no heterogomph spinigers present	*Neanthes kerguelensis* (McIntosh, 1885)
6	Conical paragnaths in Area VI, single sparse row of paragnaths in Area VII–VIII (sometimes absent); falcigers present in dorsal fascicle of neuropodia	*Nereis eugeniae* (Kinberg, 1865)
–	Shield-shaped bar in Area VI, more than 1 row of paragnaths in Area VII–VIII; falcigers absent from dorsal fascicle of neuropodia	*Perinereis atlantica* McIntosh, 1885, comb. n.*

* The single record from the Falkland Islands (Prat 1898) is considered doubtful

## Supplementary Material

XML Treatment for Gymnonereis

XML Treatment for Gymnonereis tenera

XML Treatment for Eunereis

XML Treatment for Eunereis patagonica

XML Treatment for Neanthes

XML Treatment for Neanthes kerguelensis

XML Treatment for Nereis

XML Treatment for Nereis eugeniae

XML Treatment for Perinereis

XML Treatment for Perinereis atlantica

XML Treatment for Perinereis falklandica

XML Treatment for Platynereis

XML Treatment for Platynereis magalhaensis
